# From the day they are born: a qualitative study exploring violence against children with disabilities in West Africa

**DOI:** 10.1186/s12889-018-5057-x

**Published:** 2018-01-17

**Authors:** Janet Njelesani, Goli Hashemi, Cathy Cameron, Deb Cameron, Danielle Richard, Penny Parnes

**Affiliations:** 1 Department of Occupational Therapy, NYU Steinhardt School of Culture, Education, and Human Development, Pless Hall, 82 Washington Square East, 6th Floor, New York, NY 10003 USA; 2 Samuel Merritt University, Oakland, USA; 3 The University of Toronto, Toronto, Canada

**Keywords:** Disability, Qualitative, Child protection, Guinea, Niger, Sierra Leone, Togo

## Abstract

**Background:**

Despite the building evidence on violence against children globally, almost nothing is known about the violence children with disabilities in low and middle-income countries (LMICs) experience. The prevalence of violence against children with disabilities can be expected to be higher in LMICs where there are greater stigmas associated with having a child with a disability, less resources for families who have children with disabilities, and wider acceptance of the use of corporal punishment to discipline children. This study explores violence experienced by children with disabilities based on data collected from four countries in West Africa- Guinea, Niger, Sierra Leone, and Togo.

**Methods:**

A qualitative study design guided data generation with a total of 419 children, community members, and disability stakeholders. Participants were selected using purposive sampling. Stakeholders shared their observations of or experiences of violence against children with disabilities in their community in interviews and focus groups. Thematic analysis guided data analysis and identified patterns of meaning among participants’ experiences.

**Results:**

Results illuminate that children with disabilities experience violence more than non-disabled children, episodes of violence start at birth, and that how children with disabilities participate in their communities contributes to their different experiences of violence.

**Conclusions:**

The study recommends policy-oriented actions and prevention programs that include children and their families in strategizing ways to address violence.

## Background

Violence exists in every country of the world, cutting across culture, class, education, ability, income and ethnic origin and therefore can occur against any child, but children with disabilities are at a significantly greater risk than their non-disabled peers [1, 2]. In this study violence is defined broadly as any act or threat of sexual, physical, or psychological abuse, neglect, or maltreatment. Evidence from a systematic review found that children with disabilities experience violence four times more frequently than non-disabled children in high income countries [3]. This systematic review noted the lack of systematically collected data from low and middle-income countries (LMICs), pointing to an underrepresentation of the issue of violence against children with disabilities in LMICs on the agendas of governments and researchers [4]. Despite a dearth of evidence, the prevalence of violence against children with disabilities can be expected to be higher in LMICs where there are greater stigmas associated with having a child with a disability, less resources for families who have children with disabilities, and wider acceptance of the use of corporal punishment to discipline children [5]. In addition, rates of violence against children are greater in regions with high levels of social conflict, war, crime, domestic violence, unemployment, and poverty [6].

Although there is building evidence on violence against children globally, almost nothing is known about the violence children with disabilities in LMICs experience, even though there are approximately 93 million children living with disabilities in LMICs [7] and research into the prevention and treatment of violence against children has been outlined as a global priority [8, 9]. The phenomenon remains largely undocumented due to a variety of reasons, including the fact that in some countries some forms of violence against children with disabilities may be socially accepted, not perceived as being abusive, or incidences are considered to be family affairs [10]. Furthermore, there are a lack of central agencies at the country or regional levels that gather, collate, and disseminate data about violence against children with disabilities. Of the existing data, at best it can be considered an estimate since violence against children and particularly those living with disabilities is all too often unreported. Many children are afraid to report incidents of violence against them and there may be no safe or trusted ways for children or adults to report the violent acts. From studies conducted in Kenya, Uganda, and Tanzania, children with disabilities were found to be more likely to be violated compared to their nondisabled peers [11]. In a case study of violence against children in Kenya, having mental health impairments or needing emotional or physical support were found to be risk factors for violence [10].

Understanding the violence occurring against children with disabilities is an essential first step in developing effective prevention programs to meet the rights of children. To address the dearth of information about violence that children with disabilities experience, this study explored violence against children with disabilities in the West African countries of Guinea, Niger, Sierra Leone, and Togo from the perspectives of children, community members, and disability stakeholders.

While the attention to violence against children with disabilities is scant in these four countries, some evidence exists on violence against children broadly. No cross-country or cross-cultural study concerning these four countries has previously been carried out; however, available evidence suggests that children in these countries overall experience high rates of violence. For example, the use of violent discipline in the home (defined as percentage of children who experienced any violent discipline /psychological aggression and/or physical punishment in the past month) was found to be 82% in Niger, 82% in Sierra Leone, and 93% in Togo, with no data available for Guinea [9]. UNICEF has also documented that violence against children takes many different forms in West Africa, with violence based on cultural beliefs and gender norms being widespread, physical violence and sexual abuse found to be common in schools, institutions, and homes, and with the majority of violent acts perpetrated by people who are part of children’s lives; parents, teachers, and peers. It is also noted that in West Africa violence against children is widely accepted as a form of discipline and considered to be an internal family matter, which is dealt with by the family or community without recourse to social or judicial services in order to maintain community harmony [8].

## Methods

A qualitative descriptive study was conducted in the countries of Guinea, Niger, Sierra Leone, and Togo as part of a larger study entitled “Development of a Regional Framework to Empower Children with Disabilities to Access their Right to Education and to Protection in West Africa” funded by Plan International [12]. The study received ethical approval from the University of Toronto and the governments of Guinea, Niger, Sierra Leone, and Togo. Several complementary methods of data collection were used to ensure that the findings would be comprehensive and provide both breadth and depth of information. Data collection included key informant interviews and focus groups. Interview and focus group questions were developed from the child protection literature reviewed. Local research assistants reviewed these guides and made suggestions about appropriate modifications to be made for each country. The interview and focus group guides were then revised in each country following a preliminary analysis to gain a richer understanding of the information that had been discussed in previous data collection sessions.

A purposeful recruitment strategy was utilized to recruit participants from the following three groups in order to understand the diverse experiences of stakeholders and ensure representation across rural/urban communities, gender, ethnicity, and social class:

***Group 1:*** Disability stakeholders representing local, national, or international organizations that provide support to children with disabilities, and representatives of governmental bodies responsible for disability legislation and policy.

***Group 2:*** Community members including parents, teachers, and community leaders (e.g., village chief, church clergy).

***Group 3:*** Children (10 years of age or older) with or without a disability.

Recruitment of participants was facilitated through Plan International and the international and local research team’s existing relationships with local, national and international NGOs, government ministries and other organizations working in the disability and/or child protection fields and through word of mouth and snowball sampling. Potential participants were contacted by telephone, email, text message, or in person. At that time they were explained the purpose of the study, how the interview or focus group would be conducted, and all questions were answered. If an individual was interested in participating, a mutually agreed upon date, time, and location was arranged for the interview or the individual was informed about a predetermined date for a focus group discussion.

Data collection occurred at locations that were convenient and agreed to by the participants and accommodated the need for privacy and confidentiality (e.g., office space of NGO with disability stakeholder, space provided at after-school program with child). Before each session, a researcher reviewed the consent form with the participant, clarified any questions or concerns, and obtained consent from each participant. For child participants, parents or guardians were approached first by a researcher to explain the purpose of the study and participation requirements and then asked to provide consent for their child’s participation. Interviews and group discussions lasted 30 to 90 min and were digitally audio-recorded. Individuals participated in either a one-on-one interview or a focus group discussion, but not both. Participants were offered the opportunity to have the interview conducted in English, French, or their local language.

Teams of local research assistants were recruited and trained in each country to work with the international researchers. The local researchers were recruited from local Disabled Peoples Organizations (DPOs), research organizations, and colleges with expertise in disability. The research assistants were selected based on their linguistic and cultural knowledge, qualitative research interests, as well their knowledge of disability in their country. Training in each country included reviewing the study protocol, data collection tools, and ethical guidelines. The training also include role playing interviews and focus groups, and piloting the interview and focus group guides. International researchers worked closely with the local research assistants and engaged in reflexive practices to critically analyze the process and implementation of data collection throughout the study in order to identify, discuss, and mitigate potential group or individual-level vulnerabilities that may have emerged among participants.

Clarke and Braun’s [13] approach to thematic analysis guided data analysis and identified patterns of meaning among participants’ experiences. Using this approach, thematic analysis included first reading through the transcripts to gain familiarity. Then, initial codes were generated by organizing the data into meaningful groups. Next, the researchers searched for themes by sorting coded extracts into similar ideas and themes were reviewed through several discussions with all research team members to refine the themes and narrow their focus. Theme names were then generated through collaborative discussions among team members and reviewing participant quotes. Finally, through the report writing process, connections among various quotes and codes were made clearer to produce final themes and theme names. To enhance the trustworthiness of the study, participant quotes were used to reflect their perspectives and the final themes were confirmed among all members of the research team via investigator triangulation.

## Results

In total, 419 participants participated in interviews (*n* = 189) or focus groups (*n* = 230) across the four countries (see Table 1). Despite this study being qualitative in nature and qualitative studies generally not having such large sample sizes, data generation continued until we had 419 participants, as it was at that time we had a rich and full data set that sufficiently grounded and conceptualized the issues across the four countries.

**Table 1:** Participants Recruited in each Group by Gender and Country of Study

	Country	Women/Girls	Men/Boys	TOTAL
Group 1	Guinea	3	7	10
	Niger	4	11	15
	Sierra Leone	5	18	23
	Togo	3	3	6
	Total	15	39	54
Group 2	Guinea	2	8	10
	Niger	11	21	32
	Sierra Leone	20	44	64
	Togo	6	20	26
	Total	39	93	132
Group 3	Guinea	26	36	62
	Niger	25	28	53
	Sierra Leone	46	59	105
	Togo	6	7	13
	Total	103	130	233
TOTAL		100	191	419

West Africa does not represent a single culture or society and includes 16 countries—only four were included in this study. However, key communal concerns were identified that occurred across participants’ groups, data collection methods, and the four countries. Participants indicated that violence begins the day children with disabilities are born, is very common for children with disabilities, and is experienced differently depending on how children participate in their environments. These concerns were all identified in all four countries, although the nature, range, and experiences of violence varied depending on context. For example, in post-conflict Sierra Leone, some of the described incidents of violence of children with disabilities were specific to conflict settings (e.g., children in wheelchairs were left behind during the war and killed because they could not be carried) and were not mentioned in the other countries.

### From the day they are born

Amongst the countries studied, participants cited beliefs held about children who are born with impairments that put children with disabilities more at risk of violence from the day that they are born than non-disabled children. Common beliefs cited across the four study contexts included that children born with impairments are a punishment and/or curse from God, the consequence of an undesirable act done by a parent or close relative, a curse or witchcraft, or the reincarnation of an ancestor. The following quotes illustrate these beliefs:

*Families have inherited something, a curse on the family, maybe the father has done something on other generations* (local council representative, Sierra Leone, male).

*When someone gives birth to a child with polio or who is blind, they will say “this is the devil”* (mother of a child with a disability, Sierra Leone, female).

*Disability can be caused by an evil curse or fate. A child with a disability is a real burden on the family. There are some women who would wish that their disabled child dies instead of surviving* (mother of a child with a disability, Niger, female).

*Many people continue to believe that people with disabilities are a curse, a punishment from God, a result of witchcraft or that their parents are wizards, demons* **(**institutional stakeholder, Guinea, male).

In some cases, these perceptions of children with disabilities as bizarre, supernatural, and demons have led people to believe that children with disabilities do not deserve to live. Participants across all four countries reported practices that involve killing children who are born with impairments. Such practices were reported as no longer customary in the majority of communities; however, the participants mentioned that such behaviors are still practiced in certain regions, especially in rural communities. Such behaviors were described:

*Before, there were many things being told about children with disabilities, especially those with severe disabilities such as children with cerebral palsy who cannot stand. In my community, these children are called snakes. We call these children snake because they lie on the ground. To eliminate (kill) the child, ceremonies are organized at the river, where the child is left to drown and it is said that the snake is gone and certain ceremonies prevent the return of the snake in the family* (local NGO representative, Togo, male).

*When a mother has a disabled child the father and the community say that this child is a witch. At midnight, they take the child into the bush, kill it and leave it there. In the morning, they tell the community that the witch has returned to where it came from* (community member, Sierra Leone, male).

*Some are abandoned in the woods because it is believed that the spirits will come and take them. We must leave them at the foot of a tree because the spirits will pick them up. Other practices include giving them potions: if he survives, he is human, if not, they think he is a curse* (institutional stakeholder, Guinea, female).

### All too common

Almost all the children with disabilities interviewed reported experiencing some form of violence from parents, teachers, peers, or community members. The type of behaviors that were reported included teasing, bullying, restricted food access, and physical punishment. The children with disabilities attributed these provocations to their disability, as the perpetrator often made reference to the child’s disability when carrying out the maltreatment. For example, children stated: *They will beat us just because of our disability* (child with a disability, Guinea, male) and *The other children berate me and say that I am only a half-person* (child with a disability, Guinea, female*).*

Parents and community workers also reported widespread maltreatment that children with disabilities face from a range of community members:

*Those who are lucky enough to survive are victims of prejudice. They are oppressed, abused by their parents (hidden, isolated, eating alone in dirty dishes or barely fed, poorly maintained), discriminated by their peers, merchants, pregnant women (out of fear that their unborn child will be contaminated), finally from the whole community* (community member, Togo, male).

*Children with disabilities are victims of several types of abuse: they are beaten in the streets, they are ridiculed and rejected not only by their peers but also by some adults* **(**community leader, Guinea, female).

### Differences in impairment and participation

Participants across the study contexts spoke of how violent experiences varied according to the type of impairment and the child’s participation in their community. Participants reported that children with visual, communicative, and cognitive impairments experience the most violence, while children with physical impairments experience less:

*The greatest discrimination is towards those with mental deficiencies, the blind, the epileptic. Their disability is considered like a contagion* (child with disability, Niger, male).

*The categories of disabilities best treated are those who have problems in the limbs because we are more interested in them because they can be more profitable in a trade for example, compared to others who have vision or mental problems* (community leader, Guinea, female).

*Discriminatory behavior varies according to the type of disability. With motor disabilities, there are different degrees. For me, for example, it is not too serious, but there are those who stay on the ground. We understand that these people are more discriminated against than those who can walk. Abuse is also more pronounced for the mentally handicapped* **(**Disabled Persons Organization, Togo, male).

Some participants believed children with communication impairments face greater risks as they cannot speak out or tell of their abuse:

*The ones I heard of, these children are really beautiful and mostly hearing and speech that are most vulnerable to abuse….. you just see the child pregnant* **(**education administrator, Sierra Leone, female).

The belief that children with physical impairments experience less violence was believed to be especially true for children with less participation restrictions. For example, children who can independently access their environment with the use of a mobility aid such as a tricycle were thought to receive less maltreatment. Some participants believed that the differences in the treatment of children with disabilities could be attributed to the greater participation of children with physical impairments in society, such as going to school:

*There is no discrimination against physical disabilities, unlike mental disabilities. Children with physical disabilities should go to school, although it depends on the disability. If a child can get to school by himself, he should attend. But a mentally disabled child, or one that has no control over his movements, should be treated first before we can think about putting him in school. Nobody is against the education of children with disabilities – it’s a just matter of common sense* (community leader, Niger, female).

*In general, children who do not have a heavy disability go to school. When the child has no difficulty with mobility, he can be enrolled in school, when he does not suffer from speech or impairments or from serious mental disorders he can be enrolled in school* (traditional leader, Togo, male).

## Discussion

This is the first qualitative study to explore the experiences of violence against children with disabilities in Guinea, Niger, Sierra Leone, and Togo from the perspectives of children with disabilities, their community, and disability stakeholders. The study revealed the pervasive nature of violence against children with disabilities and is consistent with findings from the limited number of studies published on violence against children with disabilities in West Africa [14–16].

As a result of violence being *all too common*, some children with disabilities are kept at home and not attending school. Parents described the need to protect children by keeping them at home since schools are often perceived to be unsafe. In these cases, children with disabilities are not receiving the benefits of an inclusive educational setting such as increased academic achievement, peer acceptance, and self-esteem, a richer friendship network, and positive lifetime benefits (higher salaried jobs, independent living) [17]. Understanding violence as a barrier to school attendance and performance is crucial for understanding how to improve the inclusion of children with disabilities.

*From the day they are born* highlights the ongoing practice of infanticide against children born with impairments. When a child is born with an impairment, the family may not understand what caused it to happen and it may be attributed to God or an unknown force [18]. This view of a child as a non-human spirit is influenced by West African religion and animism and may lead to killing of children with impairments [19]. Some of these reported experiences of violence have been described in earlier studies focusing on ritual killing of children born in West Africa and noting that children with a “body difference” were killed, but a dearth of information exists as the practice is not officially observed and records of death from infanticide are not often available. Therefore, the practice may be more widespread than formally documented [14]. This study provides evidence that the practice of ritual killing of children with disabilities exists in all four countries studied, particularly in small villages or remote communities, but is not as common in cities where detection of killings by officials is more likely to occur.

The idea that *differences in impairment and participation* of the child is a cause for violence, shifts the burden of the violence to the child. This idea is similarly seen in the broader child violence literature when an adult’s own accountability as a possible perpetrator of violence is removed by shifting blame to the child and their personal factors [20]. But no violence against children is justifiable. The circumstances that lead to violence are myriad and complex and cannot rest solely on the child. Stigma associated with disability, attitudes and traditional beliefs about disabilities, and the perception that children with disabilities are unworthy, combined with traditional gender norms, help explain why children with disabilities are at greater risk of violence in the communities studied [21].

### Limitations of the study

While a varied sample was reached, a limitation of the study was the inability to access remote villages beyond the provincial capital in some of the countries, particularly Guinea. During the course of the study, some participants in each country noted that some families in rural areas conceal their disabled children from the community, as it is relatively easier to do so in such areas rather than in the more populous urban areas, so the experiences of these families may not have been captured.

Another limitation of the study was the unequal gender representation among participants. There was a greater representation of boys/ men than of girls/ women. This was mainly due to the composition of participants in the community groups. While efforts were made to have greater female representation among participants, the research team was unable to recruit comparable numbers of women/girls and men/boys.

## Conclusion

The qualitative study presented in this article describes the violence experienced by children with disabilities in Guinea, Niger, Sierra Leone, and Togo from the perspectives of children, community members, and disability stakeholders. The study contributes to the literature on violence against children with disabilities, which in West Africa is largely nonexistent. Findings illuminate that violence against children with disabilities needs to be addressed as they experience violence more than non-disabled children from birth.

In light of the findings in this paper, future national initiatives across Guinea, Niger, Sierra Leone, and Togo, could include: (i) Increasing awareness of existing legislation and policies (e.g., UNCRPD, UNCRC) related to children with disabilities at the regional and local levels. In Sierra Leone, the Ministry of Social Welfare, Gender and Children’s Affairs in collaboration with UNICEF has a Braille version of the Child Rights Act and provides training to blind children. These children have reported that they felt empowered and more confident to advocate for their rights as they could understand the Child Rights Act in their own communication medium. (ii) Prohibit corporal punishment of children in all settings. Togo is the only country that has currently fully prohibited corporal punishment of children in all settings. (iii) Develop partnerships and coalitions with civil society institutions, NGOs, and DPOs to more effectively advocate and influence policy and programming. The relevant organizations and networks to consider for partnerships would vary by country but could include: UNICEF, CBM, Handicap International (HI), DPOs and Leonard Cheshire. In addition, it is good practice to develop relationships with representatives across all Ministry Departments, particularly education, disability, health, and protection. In Niger, HI has collaborated extensively with the Ministry of Education, Special Education Division to develop a training manual on disability issues.

## Abbreviations

CBM: Christian Blind Mission;
CRC: Convention on the Rights of the Child
CRPD: Convention on the Rights of Persons with Disabilities
DPO: Disabled Persons Organization
HI: Handicap International
UN: United Nations
